# Evolution of Quantitative Optical Coherence Tomography Angiography Markers with Glycemic Control: A Pilot Study

**DOI:** 10.3390/biomedicines10102421

**Published:** 2022-09-28

**Authors:** Thibault Ruiz, Anne Dutour, Danièle Denis, Alban Comet, Martin Eisinger, Marie Houssays, Patrice Darmon, Sandrine Boullu, Astrid Soghomonian, Thierry David, Bénédicte Gaborit, Pierre Gascon

**Affiliations:** 1 Department of Ophthalmology, Aix-Marseille University, Hopital Nord, Chemin des Bourrely, 13015 Marseille, France; 2 Department of Endocrinology, Metabolic Diseases and Nutrition, Pôle ENDO, APHM Assistance Publique Hopitaux de Marseille, 13015 Marseille, France; 3 Institut National de la Santé et de la Recherche Médicale (INSERM) 1263, Aix Marseille University, INSERM, INRAE, C2VN, 13005 Marseille, France; 4 Centre Monticelli Paradis, 433 Bis Rue Paradis, 13008 Marseille, France; 5 Groupe Almaviva Santé, Clinique Juge, 116 Rue Jean Mermoz, 13008 Marseille, France

**Keywords:** diabetes, optical coherence tomography angiography, diabetic retinopathy, diabetic maculopathy

## Abstract

**Aim:** We aimed to analyze changes in retinal microvascularization with intensive reduction of glycated hemoglobin A1c (HbA1c) in patients with poorly controlled diabetes using quantitative optical coherence tomography angiography (OCT-A) metrics. **Method**: This was a retrospective observational study in patients with uncontrolled diabetes admitted to the hospital for glycemic control. A second set of 15 healthy volunteers was included to serve as a control group. OCT-A was performed at inclusion and at 3 months to measure foveal avascular zone area (FAZA), vessel density (VD) of the superficial capillary plexus (SCP) and deep capillary plexus (DCP), acircularity index (AI), and fractal dimension (FD). **Results**: This analysis included 35 patients (35 eyes): 28 type-2 diabetics and 7 type-1 diabetics. Mean HbA1c was 13.1 ± 2.0% at inclusion and 7.0 ± 1.5% at 3 months. In the short period from inclusion to 3 months post-inclusion, patients showed significant decrease in VD–DCP (28.8% vs. 27.8%; *p* = 0.014), a significant increase in FAZA (0.300 mm^2^ vs. 0.310 mm^2^; *p* < 0.001), and a significant increase in AI (1.31 vs. 1.34; *p* < 0.01). Multivariate analysis found an increase in FAZA was correlated with baseline HbA1c level and age (R^2^ = 0.330), and a decrease in VD-DCP was correlated with HbA1c decrease and diabetes duration (R^2^ = 0.286). **Conclusions**: Rapid glycemic control in patients with uncontrolled diabetes led to possible short-term microvascular damage that correlated to both initial and decreased HbA1c.

## 1. Introduction

Diabetes is a chronic condition that affects over 400 million people worldwide [1] and causes a number of vascular complications, including diabetic retinopathy. The gold standard for diagnosis of diabetic retinopathy is dilated fundoscopic examination by slit-lamp biomicroscopy to find signs of microaneurysm, hemorrhages, and/or neovascularization. Fluorescein angiography is a more sensitive technique for screening early-stage diabetic retinopathy, but it is invasive, expensive, and has potential adverse effects.

Optical coherence tomography angiography (OCT-A) is a rapid and non-invasive examination scan that detects contrast in motion signals and compares the signal variance due to red blood cell movement. OCT-A can detect microvasculature abnormalities earlier than fundoscopic examination [2], and OCT-A can predict the risk of developing DR and can detect the onset of diabetic macular oedema [3]. A number of OCT-A metrics correlate to progression of DR and enable more reliable clinical monitoring than fluorescein angiography [3].

A large body of evidence has demonstrated that, compared to standard care, intensive glycemic control therapy designed to lower blood glucose levels reduces the risk of development and progression of DR over several years in type 1 (T1D) and type 2 diabetes (T2D) [4]. Achieving glycemic control to near-normal blood glucose levels is therefore relevant to prevent the development of DR. Risk factors for the development and progression of diabetic retinopathy (DR) include poor glycemic control, long duration of diabetes, and poorly controlled blood pressure [5]. However, these studies also found a paradoxical effect called ‘early worsening of diabetic retinopathy’ (EWDR), which manifested in around 10% of T1D [6] and T2D [7] patients with diabetes. In these patients, DR worsened rapidly within 18 months of a sharp, intensive drop in blood glucose levels. Identifying individuals at risk of EWDR is crucial in order to improve the management of newly diagnosed, poorly controlled patients with diabetes whose compliance to ophthalmologic follow-up has proven to be poor [8].

The mechanisms underpinning EWDR remain largely unknown. OCT-A-based analysis by Lavia et al. (2020) [9] found loss of vessel density at the superficial retinal plexus and deep retinal plexus. The course of change in retinal microvascularization following rapid glycemic control in patients without EWDR remains unknown.

To address this gap, in this study, we used OCT-A to assess the course of retinal microvascularization in patients with poorly controlled diabetes and without EWDR in response to intensive rapid glycemic control compared to healthy controls.

## 2. Materials and Methods

### 2.1. Study Population

This retrospective, observational, single-center study (University Hospital—Marseille North) was approved by the Marseille public hospitals system institutional review board (RGPD/APHM register 2021/1, PADS20-304). This research was conducted in full compliance with Declaration of Helsinki principles and practice. All patients and healthy subjects provided written informed consent to participate in the study.

Inclusion criteria were: any patient aged over 18 years old with poorly controlled diabetes, defined as glycated hemoglobin A1c (HbA1c) ≥ 9% (75 mmol/mol) [10] hospitalized to the endocrinology department at Marseille North University Hospital (France) for intensive glycemic control. Intensive glycemic control was defined as 2% reduction in HbA1c at 3 months (follow-up visit). Exclusion criteria were: presence of proliferative or severe non-proliferative DR, macular oedema, other (non-DR) retinopathy, previous intraocular surgery performed in the 12 months prior to inclusion, any previous history of intravitreal injection (IVI), any vitreoretinal interface disorders preventing a good-quality OCT-A scan (i.e., with a signal strength index (SSI) of ≥7), axial length of the eye greater than 26 mm, a refractive error of more than +3 diopters (D) or less than −6D, or more than +2D cylinders, previous history of peripheral pan-retinal photocoagulation, uncontrolled hypertension, recent history of bariatric surgery, controlled diabetes, and poorly controlled diabetes with HbA1c < 9% (75 mmol/mol).

A control group (15 healthy volunteers) with normal fasting plasma blood glucose levels (<126 mg/dL, 7 mmol/L) and no antidiabetic treatment was analyzed in parallel.

### 2.2. Clinical Assessment

Full patient interviews and eye exams were completed for all subjects, and the questionnaire EPICES [11] (Evaluation of the Deprivation and Inequalities of Health in Healthcare Centers) was collected at admission. Age, gender, diabetes duration, anti-diabetic medications, and diabetic complications were recorded. All patients underwent a general clinical examination in-hospital to screen for micro and macrovascular complications of diabetes, arterial hypertension, and dyslipidemia. All patients received at least one full blood test, including glycated hemoglobin and serum creatinine assay. Therapeutic intensive glycemic control was recorded (intravenous insulin therapy, continuous subcutaneous insulin infusion (CSII), glucagon-like peptide-1 receptor agonist (GLP-1 RA), and/or basal bolus regimen introduction) according to current diabetes clinical practice guidelines [12].

Hypo- or hyperglycemic episodes between inclusion and follow-up visits were systematically recorded, and subsequent antidiabetic treatment adjustments were collected for each patient. All patients underwent ophthalmological examination at inclusion and at 3 months that included slit-lamp biomicroscopy, fundus photography, and OCT-A.

### 2.3. Imaging Protocol

Fundus photography was done using a widefield imaging device (Optos Daytona, Optos PLC, Dunfermline, UK), and OCT-A with Cirrus 6000OCT-Angiography scans (Zeiss OCT, Carl Zeiss Meditec, Jena, Germany) with 3 × 3 mm and 6 × 6 mm foveal-centered scan patterns at an acquisition frequency of 100,000 A-scans per second, a wavelength centered on 840 nm, and axial and transverse resolution of 5 µm and 12 µm, respectively. Motion signal contrast was captured using complex-based OCT-microangiography (OMAG) technology software that coanalyses both signal phase and signal intensity. All images were produced by a single appropriately trained operator (‘TR’). The built-in device software segmented the superficial retinal plexus and deep retinal plexus, and segmentation was checked after each image acquisition and manually corrected if necessary.

Any OCT-A images presenting significant artefacts (motion, projection, doubling of the retinal vessels, stretching defect, etc.) were excluded.

Furthermore, all images with an SSI < 7 were excluded. The workflow for all images used eye-tracking software, a signal motion correction algorithm, and an integrated projection artefact correction tool.

DR staging was conducted by two skilled ophthalmologists (‘TR’ & ‘PG’) working blind on the ultra-widefield imaging and was based on the Airlie House classification system [13].

### 2.4. Post-Processing Workflow

Vessel density (VD) was defined as the percentage of a given area that was occupied by blood vessels for both the superficial and the deep capillary plexus.

The foveal avascular zone (FAZ) is a region at the center of the retina that lacks retinal blood vessels.

Fractal dimension (FD) is a measure used to analyze the complexity of a structure, with higher values indicating higher complexity.

The acircularity index (AI) represents the circularity defect of a given region.

**Figure 1:**
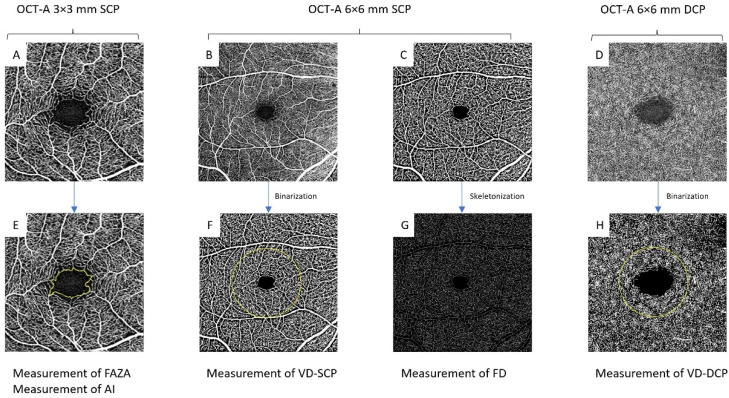
Roll-up summary of the measures performed: (**A**): 3 × 3 mm OCT-A imaging used to measure FAZA and AI (**E**); (**B**,**C**): 6 × 6-mm OCT-A images of the superficial capillary plexus (**B**) before and (**C**) after binarization to measure (**F**) vessel density and (**G**) fractal dimension after skeletonization; (**D**): 6 × 6-mm OCT-A images of the deep capillary plexus to measure vessel density (**H**) after binarization. FAZA: foveal avascular zone area; FD: fractal dimension; DCP: deep capillary plexus; VD: vessel density; AI: acircularity index; SCP: superficial capillary plexus.

Post-processing analysis was performed after exporting the image to image J (National Institutes of Health (NIH), Bethesda, MD, USA) via a workflow that involved converting en face angiograms of the superficial and deep capillary plexus into 8-bit format, then binarizing (using the Niblack method for the superficial capillary plexus, as previously described [14]) and skeletonizing the image with all vessels converted to the diameter of a single pixel. For the deep capillary plexus, we used a custom binarization method with thresholding at 128 for white pixels and 0 for black pixels to eliminate binarization noise from the foveal avascular zone (FAZ) (Figure 1).

The same investigator (‘TR’) ran blind analysis on all the images exported from the workflow to measure the area and acircularity index of the FAZ and to measure vessel density on the middle 3 mm of the binarized image. Fractal dimension was measured on the skeletonized image using the box-counting method in Fractalyse software (ThéMA, Besancon, France). For the deep capillary plexus, we only analyzed vascular density. Acircularity index was calculated using the equation: $\mathrm{AI}=\frac{\mathrm{PFAZ}}{2\pi \frac{\sqrt{\mathrm{FAZA}}}{\pi }}$, where PFAZ is perimeter of the FAZ.

### 2.5. Statistical Analysis

Statistical analysis was performed using SPSS software suite v26.0 (IBM Corporation, Armonk, NY, USA). Results are reported as mean ± standard deviation (±SD) or as number and percentage (%). Statistical significance was set at *p* < 0.05.

In this study, we used an intra-class correlation coefficient to test for reproducibility of the measurements. Inter-eye correlation was measured using the Pearson correlation coefficient to ascertain whether to use one or both eyes. A paired *t*-test was used for comparison of retinal parameters at baseline vs. 3 months.

Multivariate analysis used multiple logistic regression for quantitative data and ANOVA with Fisher’s F-test for qualitative data.

## 3. Results

### 3.1. Patient Demographics

**Figure 2:**
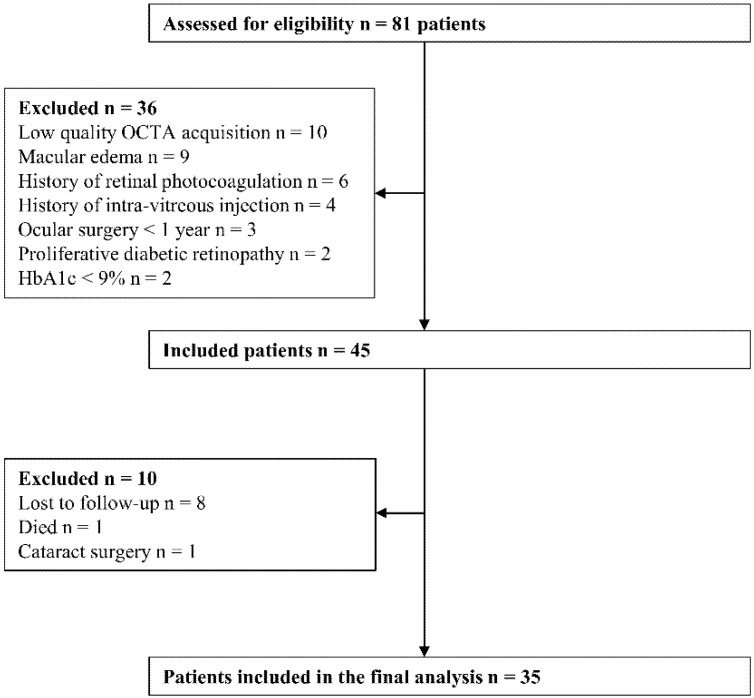
Flowchart of patient eligibility.

The study included a total of 35 eyes from 35 patients with diabetes (Figure 2) and 15 eyes from 15 healthy volunteers.

The mean age of the patients with diabetes was 51.1 ± 2.3 years old (Table 1). The mean age of the healthy control subjects was 40 ± 10.4 years old.

Mean HbA1c in the group with diabetes was 13.1 ± 2.0% (120 ± 1 mmol/mol) at baseline and 7.0 ± 1.5% (53 mmol/mol) at 3 months, for a mean diabetes duration of 3.6 ± 1.0 years. Hypertension and dyslipidemia were present in 51% and 74% of patients, respectively. Sixty percent of patients (21/35) were hospitalized for newly discovered diabetes, 9 had acute complications of diabetes: 2 had diabetic ketoacidosis (DKA) and 7 had hyperglycemia with ketosis alone. Seven patients had a mean duration of disease < 5 years, 3 were between 5 and 10 years, and 4 patients had a mean duration > 10 years. Four out of five patients (80%) had type-2 diabetes, 23/35 (66%) took no antidiabetic drugs at baseline, 8/35 (23%) were already on oral diabetes medication, 3/35 (8.6%) were on subcutaneous multi-daily insulin injections, and one patient had combined both oral diabetes medication and basal insulin. DR was found in 10/35 patients (28.5%): staged as mild non-proliferative DR in 8 patients (22.8%) and moderate non-proliferative in 2 patients (5.7%). Mean EPICES score was 3.1 ± 1.7 in the population included in this analysis vs. 4.7 ± 1.4 in subjects lost to follow-up (*p* = 0.03).

Intensive and immediate glycemic control was obtained by intravenous insulin in 10 patients (29%), CSII in 4 patients (11%), GLP-1 RA in one patient (3%), and basal bolus regimen in 20 patients (57%). At hospital discharge, 11 patients had GLP-1 RA (31%) introduction and 2 patients an SGLT2 inhibitor. At the follow-up visit, insulin therapy was stopped in 8 patients. Eleven patients (31%) experienced hypoglycemic episodes. None of the patients experienced severe hypoglycemia.

### 3.2. Reproducibility of the Measurements

The intraclass correlation coefficient on reproducibility of the measurements was excellent at >0.90 for all metrics across the board. Inter-eye correlation measured using Pearson’s correlation coefficient was good-to-excellent. In the analyses that follow, we opted to use the left eye by default, as there were no missing left-eye data (there were three missing datapoints for the right eye: one excluded for macular oedema and two excluded for more than 2D refractive error) (Table 2 and Table 3).

### 3.3. Course of Quantitative Retinal Traits

Time-course changes at baseline and 3 months follow-up (M3) in VD-SCP and VD-DCP, AI, FAZA, and FD are reported in Table 4.

There was a significant increase in FAZA at baseline vs. 3 months, i.e., from 0.300 ± 0.131 mm^2^ to 0.310 ± 0.135 mm^2^ (*p* < 0.001). VD increased from 36.9% ± 1.1 at baseline to 37. 0% ± 1.2 at 3 months for SCP (*p* = 0.24) and decreased from 28.8% ± 5.7 at baseline to 27.8% ± 5. 5 at 3 months for DCP (*p* = 0.014). Furthermore, AI changed significantly between baseline (1.31 ± 0.20) and 3 months (1.34 ± 0.20; *p* < 0.01). FD did not change significantly after glycemic control, with 1.66 ± 0.01 at baseline and 1.66 ± 0.01 at 3 months (*p* = 0.44). The control group did not show any significant changes from baseline to 3 months. VD–SCP and VD–DCP were higher, and FAZA, FD, and AI were lower in the group with diabetes (Table 5).

**Figure 3:**
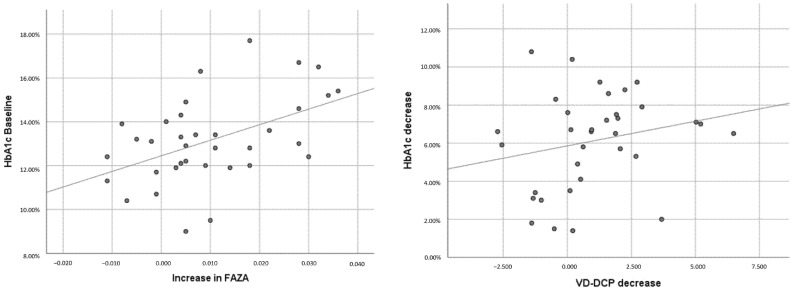
Variation in FAZA and VD-DCP plotted against baseline HbA1c and drop in HbA1c.

Multivariate analysis of FAZA, VD-DCP, and AI was made using multivariable logistic regression models adjusting for age, sex, diabetes duration, HbA1c baseline, and HbA1c decrease, and revealed that the increase in FAZA correlated with baseline HbA1c level (*p* = 0.045) and age (*p* = 0.036), while decrease in VD-DCP was correlated with diabetes duration (*p* = 0.018) and HbA1c decrease (*p* = 0.008). We found no correlation with AI in multivariate analysis. None of the variables were found to correlate with sex (Table 6, Figure 3).

### 3.4. Subgroup Analysis

Subgroup analysis based on diabetes type found a different pattern of results. A significant increase in FAZA was observed, which reached a mean of +0.014 mm^2^ from baseline to 3 months (*p* = 0.02). AI, VD–SCP, VD–DCP, and FD showed no significant differences from baseline to 3 months. In T2D mellitus patients, AI, FAZA, and VD–DCP were significantly different from baseline to 3 months: +0.04 (*p* = 0.013) for AI, +0.01 mm^2^ (*p* = 0.001) for FAZA, and −1.3% (*p* = 0.002) for VD–DCP (Table 6).

In sub-group analysis, there was no statistically significant difference in the retinal biomarkers between patients with and without the introduction of GLP1 RA therapy.

Remarkably, patients who had experienced hypoglycemic episodes between the inclusion and the follow-up visit at 3 months had a greater deterioration in IA (+0.06 vs. +0.02, *p* < 0.02) compared to patients without hypoglycemia and control patients (−0.01).

## 4. Discussion

This retrospective observational OCT-A study on retinal microvascularization evidenced that foveal avascular zone area and acircularity index increased, and vascular density in the deep capillary plexus decreased at 3 months after intensive glycemic control in predominantly newly diagnosed diabetes mellitus patients. The increase in FAZA was correlated to baseline HbA1c and age, while the decrease in VD-DCP was correlated with drop in HbA1c at 3 months and diabetes duration. There was no statistically significant change in VD–SCP and FD and no progression in the DR stage. These various quantitative OCT-A metrics have already been analyzed in several cross-sectional studies and have already been found to be markers of the course of DR [15].

FAZ area is a metric that correlates to DR stage, predicts risk of progression to a more severe DR stage, and has been very recently associated with long-term cardiovascular outcomes in people with T2D [16]. A recent meta-analysis by Zhang et al. (2021) [17] compiled and analyzed 2241 diabetic eyes and showed that FAZ area increased with worsening DR and in DR-free patients compared to healthy controls. Other older studies had already confirmed these patterns of FAZA change in both the SCP and the DCP [17].

In the UK biobank cohort, using a very recent deep-learning approach of the retina and phenome- and genome-wide analyses, Zekavat et al. showed that low retinal vascular FD and density are significantly associated with higher risks of incident mortality, hypertension, congestive heart failure, renal failure, T2D, and DR. Hence, retinal vasculature and microvascular indices could serve as biomarkers for future cardiometabolic or ocular disease [18].

There is also evidence that VD decreases with DR stage. Decreasing VD is a marker of progressive DR. VD decrease appears to onset earlier in the DCP than the SCP [19], and this earlier VD decrease metric is reported to correlate with impaired retinal function [20]. However, this could also predict the onset of macular edema [3]. This means that the decreases in VD–DCP observed here could be an early sign of progressive DR. One important thing to notice is that the data at 3 months post-glycemic control showed no decrease in visual acuity and no worsening of DR.

However, subgroup analysis only found an increase in FAZA, with no significant decline in VD–SCP, VD–DCP, or AI in T1D mellitus patients. The population analyzed in this study is too small to firmly conclude between-group differences, but it does bring evidence to further confirm previous findings. Um et al. (2020) [21] found that FAZA changed proportionally with DR stage in T1D and T2D mellitus, whereas VD–SCP and VD–DCP only declined in advanced stages of DR in T1D but declined progressively with advancing DR stages in T2D mellitus.

The prevalence of diabetic retinopathy was high in our population of recently diagnosed diabetic patients. This is consistent with a recent meta-analysis showing 25.7% of DR in the International Diabetes Foundation atlas and Eurostat population data [22]. Moreover, given the high vulnerable and precarious condition of our study population and the high level of HbA1c, the duration of diabetes was probably underestimated.

In general, the worsening of DR after rapid glycemic control is seen in patients with pre-proliferative or proliferative DR. In this study, we chose to investigate retinal parameters in patients with no vision-threatening lesions in order to introduce no bias regarding laser, IVI, or surgical treatment.

Both initial and sharply dropping glycated hemoglobin concentrations can onset or worsen DR [23]. However, little is known about the effect of these two variables on retinal microvascularization in patients that do not develop DR. To the best of our knowledge, this study uniquely marks the first analysis of changes in microvascular OCT-A features over the course of rapid glycemic normalization therapy in patients with uncontrolled diabetes. Dupas et al. (2018) [24] investigated variations in OCT-A metrics in type-1 diabetes mellitus with poorly controlled DR with and without loss of visual acuity compared to healthy controls, and found a loss of vessel density in the SCP and DCP and a relative enlargement of the FAZ. Consistent with our findings, the DCP showed more severe impairment than the SCP, especially in patients experiencing loss of visual acuity.

The physiopathology of these feature changes remains unclear and may involve an interplay of several mechanisms, including the VEGF-like effect of insulin therapy, as reviewed by Bain et al. (2019) [25]. The introduction of insulin therapy in patients who have or have not already been on hypoglycemic agents is an independent factor for worsening DR [7]. Here, all of the patients in our cohort except one had initially been treated with insulin therapy, which may possibly explain the deterioration in OCT-A metrics. The reduced-blood-glucose-induced decrease in plasma osmolarity could lead to impaired retinal vascularization and the onset of signs of retinal ischemia. Note that glycemic variability is thought to play a role in oxidative-stress-related cell death [26]. As clinical DR develops many years after the onset of histopathological lesions, these prospective OCT-A studies need to work towards screening for these lesions as early as possible by detecting their impact on vascularization. While a large-scale randomized placebo-controlled trial reported that the introduction of a GLP-1 RA (once-weekly semaglutide) significantly increased the risk of retinopathy complications (vitreous hemorrhage, blindness, or conditions requiring treatment with an intravitreal agent or photocoagulation) [27], conversely, we found no statistically significant difference regarding retinal biomarkers in the subgroup of patients for whom a GLP-1 RA had been introduced during hospitalization compared to patients treated with other antidiabetic drugs. This finding is in accordance with our previous Angiosafe DT2 study, showing no association between GLP-1 RA exposure and severe DR [28] in T2D patients. By contrast, in our study, patients experiencing hypoglycemic episodes between baseline and 3 months had significant impairment in the acircularity index compared with patients experiencing no hypoglycemia This may be attributed to the magnitude and rapidity of HbA1c reduction, as suggested by some authors [29], but this merits further long-term evaluation.

This study has several limitations. The sample size was relatively small, but the findings were based on a single eye examination, and reliability was checked between the two eyes at baseline and showed no difference at 3 months (data not shown). As this was a pilot study, no sample-size calculation could be done a priori, but further study including patients without rapid glycemic control as a control group is ongoing.

Calculated FAZA values may vary, as the measurements are done manually, but studies have found that they remain reliable [30]. Furthermore, the fact that the intra-class correlation coefficient was high and that the measurements were all performed blind by the same investigator point to good reproducibility. Furthermore, as was the case here, the artefact sensitivity of OCT-A systems leads to a large number of non-inclusions, which can create selection bias. However, in the absence of any gold standard, OCT-A remains the best way to assess retinal microvascularization.

Another bias in our study is the number of lost-to-follow-up cases. Analysis found that the subset of lost-to-follow-up patients had a higher EPICES score than the patients ultimately included in the study, which is clear proof of higher material and social deprivation. It is unlikely that these patients would have presented less decline in their OCT-A metrics, as a higher EPICES score equates to lower health literacy and therefore a greater risk of diabetes-related complications, including DR [11].

Finally, this research only used glycated hemoglobin and HbA1c reduction at short-term as a metric of poorly controlled diabetes. This limits the analysis of glycemic variability on long-term retinal microvascularization. However, AI was found to be correlated with hypoglycemic episodes in this study; future studies using sensor glucose metrics and continuous glucose monitoring are warranted to better evaluate the impact of glucose variability exposure on the retina and the risk of DR worsening.

## 5. Conclusions

This study showed a decline in several optical coherence tomography angiography metrics following intensive rapid glycemic control in patients with poorly controlled diabetes, i.e., an increase in foveal avascular zone area, an increase in acircularity index, and a decrease in vessel density of the deep capillary plexus. Further research is needed to assess the long-term course of retinal microvascularization in these patients, to evaluate the effect of rapid glycaemia control in patients with less-elevated glycated hemoglobin, and to further investigate the difference between type-1 and type-2 patients.

## Figures and Tables

**Table 1:** Initial (baseline) characteristics of the diabetic patients.

Characteristics	Value
Age	51.1 (±2.3)
Gender (male)	26 (74%)
T1D	7 (20%)
T2D	28 (80%)
Duration of diabetes (years)	3.6 (±1.0)
Baseline HbA1c (%)	13.1% (±2.0)
Hypertension	18 (51%)
Dyslipidemia	26 (74%)
Peripheral artery disease	3 (8.5%)
Chronic kidney disease	10 (28%)
Mild diabetic retinopathy	8 (22.8%)
Moderate diabetic retinopathy	2 (5. 7%)

Data are reported as mean (±SD) or number (%).

**Table 2:** Intraclass correlation coefficient on reproducibility of the measurements using Pearson’s correlation coefficient.

	FAZA	AI	VD–SCP	VD–DCP	FD
Pearson correlation coefficient	0.995	0.933	0.995	0.991	0.997
*n*	14	14	14	14	14
*p*	<0.001	<0.001	<0.001	<0.001	<0.001

**Table 3:** Inter-eye correlation using Pearson’s correlation coefficient.

	FAZA	AI	VD–SCP	VD–DCP	FD
Pearson correlation coefficient	0.948	0.879	0.868	0.837	0.855
*n*	32	32	32	32	32
*p*	<0.001	0.002	<0.001	<0.001	<0.001

**Table 4:** Patterns of variation in OCT-A metrics in diabetic patients before and after glycemic control. Data presented as mean (±SD). Significant differences are underlined and in bold.

	Baseline	M3	Δ mean	*n*	*p*
AI	1.31 ± 0.20	1.34 ± 0.20	0.03	35	<0.01
FAZA (mm^2^)	0.300 ± 0.131	0.310 ± 0.135	0.010	35	<0.001
VD–SCP (%)	36.9 ± 1.1	37.0 ± 1.2	0.1	35	0.24
FD (%)	1.66 ± 0.01	1.66 ± 0.01	0.00	35	0.44
VD–DCP (%)	28.8 ± 5.7	27.8 ± 5.5	−0.9	35	0.014

FAZA: foveal avascular zone area; FD: fractal dimension; DCP: deep capillary plexus; VD: vessel density; AI: acircularity index; SCP: superficial capillary plexus.

**Table 5:** Patterns of variation in OCT-A metrics in the control group. Data presented as mean (±SD).

	Baseline	M3	Δ mean	*n*	*p*
AI	1.28 ± 0.09	1.27 ± 0.08	0.01	15	0.54
FAZA (mm^2^)	0.284 ± 0.136	0.283 ± 0.133	0.001	15	0.53
VD–SCP (%)	37.9 ± 0.9	37.8 ± 1.0	0.12	15	0.24
FD (%)	1.67 ± 0.00	1.67 ± 0.01	0	15	0.75
VD–DCP (%)	30.3 (±4.37)	29.9 (±4.1)	−0.4	15	0.328

**Table 6:** Multivariate analysis of the evolution of FAZA, VD-DCP, and AI according to age, sex, duration of diabetes, baseline HbA1c, and decrease in HbA1c.

FAZA					VD-DCP				AI			
	Value	*p*	Confidence Interval 95%	R^2^	Value	*p*	Confidence Interval 95%	R^2^	Value	*p*	Confidence Interval 95%	R^2^
Age	0.375	0.036	(0.026; 0.724)	0.330	−0.039	0.828	(−0.398; 0.321)	0.286	−0.208	0.292	(−0.605; 0.189)	0.131
Sex	0.115	0.516	(−0.243; 0.473)		0.136	0.457	(−0.233; 0.506)		−0.122	0.545	(−0.530; 0.286)	
Diabetes duration	−0.093	0.620	(−0.471; 0.286)		**0.477**	**0.018**	**(0.086; 0.867)**		−0.036	0.865	(−0.467; 0.395)	
HbA1c Baseline	**0.589**	**0.045**	**(0.014; 1.163)**		−0.511	0.088	(−1.104; 0.081)		−0.428	0.191	(−1.082; 0.226)	
HbA1c decrease	−0.077	0.792	(−0.667; 0.513)		**0.853**	**0.008**	**(0.244, 1.462)**		0.443	0.188	(−0.229; 1.115)	

## Data Availability

All data relevant to the publication are included.
